# The Promising Role of Probiotics in Managing the Altered Gut in Autism Spectrum Disorders

**DOI:** 10.3390/ijms21114159

**Published:** 2020-06-10

**Authors:** Basma Abdellatif, Clare McVeigh, Ghizlane Bendriss, Ali Chaari

**Affiliations:** Premedical Department, Weill Cornell Medicine, Qatar Foundation, Education City, Doha, P.O. Box 24144, Qatar; bwa4001@qatar-med.cornell.edu (B.A.); clm2021@qatar-med.cornell.edu (C.M.); ghb2002@qatar-med.cornell.edu (G.B.)

**Keywords:** ASD, probiotics, gut microbiota, inflammation, dysbiosis

## Abstract

Gastrointestinal symptoms (GIS) have been reported repeatedly in people with autism spectrum disorder (ASD) and studies have reported interesting correlations between severity of behavioral and gastrointestinal symptoms. Growing evidence indicates that the gut microbiota in ASD is altered with various shifts described at different taxonomic levels, pointing to the importance of considering the gut–brain axis in treatment of these disorders. Probiotics are live beneficial bacteria that are ingested as food or customized pills. These beneficial bacteria, when added in sufficient amounts, can correct the dysbiosis. Because probiotics have shown success in treating irritable bowel syndrome (IBS), it is plausible to investigate whether they can induce alleviation of behavioral symptoms as well. Probiotics show, in some clinical studies, their potential benefits (1) in improving gastrointestinal dysfunction, (2) in correcting dysbiosis, (3) in consequently reducing the severity of ASD symptoms. This review compiles data from selected studies that investigate these benefits and the mechanisms that mediate these effects, which include the production of metabolites, hormones, and neurotransmitters and the regulation of pro-inflammatory and regulatory cytokines. Future research based on more randomized, controlled studies with a larger population size and standardized use of strains, concentration of probiotics, duration of treatments, and methods of DNA extraction is still needed in this area, which may lead to more robust results.

## 1. Introduction

Humans have developed a symbiotic relationship with a diverse group of bacteria and other microorganisms collectively known as the gut microbiota [1]. The gut microbiota is rich, with up to 100 trillion microbes that are essential to our homeostasis [2]. Initial mode of nutrition, delivery, (breast or formula) feeding, and subsequent diet can preferentially allow the dominance of one type of bacteria over the other [3,4]. The composition of the microbiota begins to stabilize between 6 and 36 months of age; during this time, it is possible to distinguish between the core microbiota and a provisional highly sensitive one [5]. This window of time is also critical for brain development as synaptic formation and myelination expand dramatically during the first few years of age [6,7,8,9]. The gut microbiota is also important for the maturation of the gut-associated lymphoid tissue; it mediates local and systemic immune responses and is therefore essential for both innate and acquired immunity [10]. Typical adult microbiota contains about 1000 different species mostly from *Bacteroidetes* and *Firmicutes* phyla [11]. Gut bacteria have shown to be critical for digesting substrates that would otherwise yield no nutrition to the host. In addition, microbes release short chain fatty acids or SFCAs that are important for the intestinal mucosa and for modulating immune response and tumorigenesis in the gut [12]. Moreover, gut bacteria are an important source for vitamin K and, to a lesser extent, vitamin B [13]. Gut microbiota dysbiosis, or the imbalance of gut bacteria composition, has been observed in cancer [14], diabetes [15,16], obesity [16,17,18], cardiovascular diseases [19], autoimmune diseases [20,21], and neurological diseases [22,23,24,25], and more importantly for the purposes of this review, autism [8,12,26,27].

Autism spectrum disorder (ASD) refers to a group of neurodevelopmental disorders that affect social interactions, communication, and repetitive behavior. The global prevalence of ASD is estimated to range from 0.1% to 1.8% [28]. Based on recent epidemiological studies, the median of prevalence estimates of ASD is 62/10,000 [29]. Current studies suggest that various factors are associated with the development of ASD, combining both genetic, epigenetic, and environmental components [30,31,32]. These factors include maternal exposure to viruses, maternal nutritional deficiencies or overloads, de novo mutations during embryonic development, paternal sperm [33], parental obesity [34], parental allergies, and dysfunctional immune systems [35]. Patients with ASD also develop limited cognitive and communication skills. Children with autism are often not able to express abdominal discomfort properly and thus gastrointestinal symptoms (GIS) are sometimes shadowed by the resulting aggression, which is in turn sometimes grouped wrongly with behavioral characteristics of autism. Signs like stool frequency, vomiting, and diarrhea are more objective and thus more helpful to research. Consequently, a reliable quantification of GIS rates in children with autism is not available but an approximation of 40% has been suggested based on the current data [36]. The causal relationship between gastrointestinal symptoms and autism is supported by studies that show frequency of GIS; chronic diarrhea and constipation tend to increase with the severity of autism. In the same context, it has been shown that irritable behaviors in ASD, like social withdrawal and anxiety, are more frequent in patients showing GIS [37,38]. Moreover, patients with ASD have shown different compositions of gut microbiota compared to the controls [39,40]. Specifically, the severity of GI symptoms in patients with ASD has been linked to the derangement of the gut microbiota [41]. This opens up further avenues of research to investigate the modulation of the gut microbiota in ASD by means of probiotics as a potentially safe therapeutic option. 

According to the Food and Drug Administration (FDA) and WHO, probiotics are defined as “live micro-organisms which can provide health benefits on the host when administered in adequate amounts” [42,43]. Probiotics may be added individually or in a mixture of different strains that work in synergy and have additive benefits [44]. The function of these probiotics varies within the same species and may exhibit different benefits when used individually or in formulation. Furthermore, these benefits may differ according to the patient group [45]. In order to substantiate the health claims and benefits of probiotic therapy, Food and Drug Organization (FAO) and WHO provide clear guidelines for an effective evaluation of the use of probiotics in food or as a supplement, as outlined in [44]. Since probiotics have been recognized for their beneficial effects on health, they have been used as potential dietary supplements [46]. In a preliminarily study using ASD mice as a model, probiotics have shown promise in alleviating some of the symptoms of autism and mood disorders by directly restoring the gut microbiota balance or by other ways such as strengthening the GI barrier through the tightening of intercellular adhesions [47]. The most common microbes used as probiotics include lactic acid bacteria (LAB) and bifidobacteria. In addition, some nonpathogenic species from the class *Saccharomyces*, *Streptococcus*, and *Lactococcus* are used [48]. Probiotics can improve the host’s health through stimulating the GI barrier function, producing antimicrobial agents, adjusting the mucosal immunity, and altering the gut’s microbiota composition [49]. Different probiotics are preferentially beneficial depending on how well they are able to survive the harsh environment of the body like the gastric acid of the stomach and the bile of the upper digestive tract [50]. In addition to overcoming these conditions, probiotics need to be metabolically active in the gastrointestinal tract while adhering to it. Furthermore, they need to be antimicrobial towards pathogenic bacteria and able to reduce the colon’s pH [51]. Even though some non-living cells have probiotic properties, it has been shown that living ones are much more effective [52]. Probiotics can be consumed in the form of a pill or through fermented foods. Karimi and colleagues conclude that probiotic bacteria viability is satisfactory at the end of typical storage periods in different kinds of cheeses including fresh, soft, semi-soft, hard, and white brined cheeses, thus confirming that cheese is a good probiotic carrier [53]. Another review shows that yogurt, fermented milk, and even fermented vegetables are also good probiotic carriers [54]. As the gut microbiota has been shown to have a bidirectional relationship with the brain, using probiotics to correct dysbiosis is a fruitful area of investigation into the improvement of neurologic diseases and neuropsychiatric disorders [55]. Since probiotics have been shown to help treat diseases with symptoms similar to those associated with neurodegenerative and neuropsychiatric diseases, more clinical research is needed to support and elucidate how they can also improve the symptoms associated with actual neurodegenerative and neuropsychiatric disorders, specifically through maintaining the bi-directional connection between the enteric and central nervous systems [56,57]. In fact, some studies have shown promise in human trials using ASD sufferers [40,58,59,60]. 

As the prevalence of ASD appears to be increasing, the need for a better understanding of the disorder and a way to treat its symptoms is pressing. The correlation between the severity of GIS and autistic behavior in children with ASD provides a promising way to tackle this disorder through correcting the gut dysbiosis. This paper aims to review published research that helped analyze the merit of using probiotics in treating autism spectrum disorder (ASD) symptoms. The paper covers gut microbiota status, GI symptoms, immune system, and inflammation in people with ASD. It also covers the results of recent research into the use of probiotics to treat ASD behavioral and gastrointestinal symptoms (GIS). 

Studies depicted in our tables were selected after searches on Google Scholar, Pubmed, and Scopus for the MesH terms: “probiotics AND autism spectrum disorder” or “gut microbiota AND ASD” filtered by years (2000 to 2020). In addition, the database clinicaltrials.gov was used to retrieve past and ongoing clinical trials. Finally, we used references of resulting selected publications when relevant. Selections were not limited by geographical location. 

## 2. The Gut–Brain Axis

**Figure 1:**
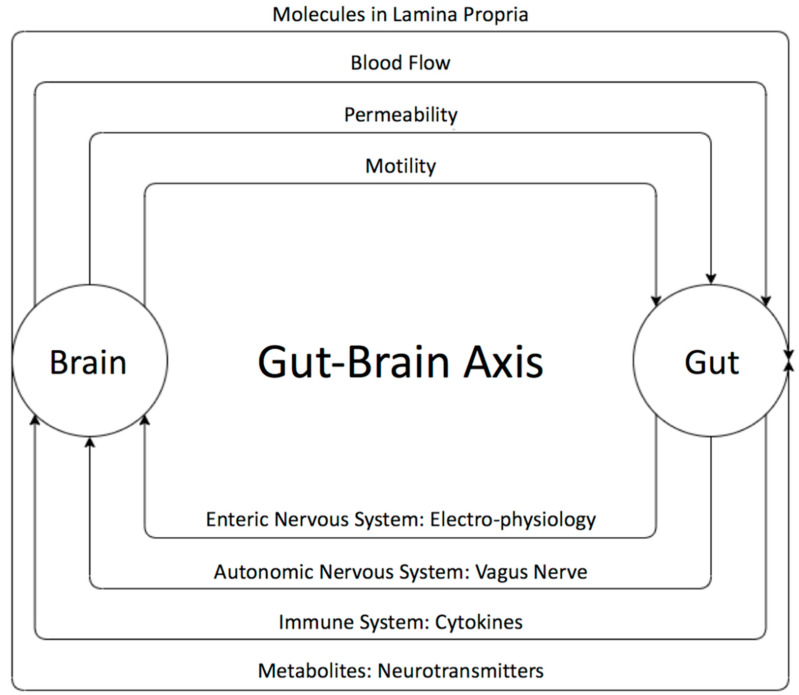
The bi-directional relationship between the gut and the brain and its players. The brain can influence the gut microbiota through changes in gastrointestinal motility, intestinal permeability, blood flow, and the release of molecules in the lamina propria. The gut microbiota, in turn, influences the brain neural as well as immune system’s communication and active metabolites. The gut microbiota affects the brain through the enteric nervous system (ENS), which communicates with the autonomic and central nervous systems.

The existence of a bidirectional control system through the gut–brain axis has been established through many studies [55,60] (Figure 1). Disturbances in the relationship between the enteric and the central nervous system are speculated to result in brain gut diseases like irritable bowel diseases. The brain can influence the gut microbiota through changes in gastrointestinal motility, intestinal permeability, blood flow, and the release of molecules in the lamina propria. The gut microbiota, in turn, influences brains functions as well as the immune system via the secretion of active metabolites [61,62]. It has been shown that these metabolites help in maintaining the health of the body through various mechanisms such anti-inflammatory activity and energy supplementation for colonic epithelium and [41]. These molecules are also shown to have a role in neuro-immunoendocrine regulation as well as contributing to various epigenetic changes [41], thereby suggesting their role in disease pathology. Some of the most important metabolites produced by microorganisms through undigested fermented food are short-chain fatty acids (SCFAs) [63,64,65,66,67,68,69]. The main SCFAs that possess neuroactive properties are acetate, butyrate, and propionate [63,64,65,66,67,68,69]. Acetate and propionate are mainly produced by Bacteroidetes, whereas Firmicutes contribute primary to the production of butyrate [42,44]. Acetate is the most abundant metabolite in the colon and stool flowed by propionate and butyrate [45]. Many studies showed the importance of SCAFs as a group of compounds derived from the host microbiome that may induce widespread effects on gut, brain, and behavior [40,47,48]. In fact, animal and human studies showed that gut dysbiosis has been implicated in behavioral and neurologic pathologies including depression, Parkinson, Alzheimer, and ASD and that microbiota manipulation and SCFA administration have been proposed as treatment targets for such diseases [49,50,65,66,67,68,69,70,71,72,73,74,75,76]. SCFAs present in the gut in an approximate molar rate of 60:20:20 for acetate, propionate, and butyrate, respectively, improve the gut health through a number of local effects, ranging from maintenance of intestinal barrier integrity, mucus production, and protection against inflammation. SCAFs can regulate the tight junction proteins like claudin-1 and occluding leading to maintenance of the integrity of epithelial barrier, thereby decreasing the “leaky gut” and consequently hindering inflammatory reactions [52]. Moreover, it has been reported that SCFAs influence the central nervous system through alteration of mitochondrial functions [53], modulation of the epigenome related to neurological diseases [54], and modulation of neurotransmitter gene expression in various types of cells [55]. In fact, SCFAs can regulate the epigenome through the inhibition of histone deacetylase (HDAC) activity, which has been found in both the gut and associated immune tissue, as well as the peripheral nervous system and central nervous system (CNS) [64]. Moreover, it has been shown that SCFAs can cross the blood brain barrier through monocarboxylate transporters and play an important role in maintaining its integrity by upregulating expression of proteins of the tight junctions. This was supported by a study showing that a germ-free mouse presented a reduction in the expression of tight junction proteins such as claudin and occluding that leads to the increase of the permeability of the blood brain barrier [77]. 

In the brain, SCFAs are able to affect glial cell function, increase neurogenesis, and maintaining neuronal homeostasis and function modulating the levels of neurotransmitters and neurotrophic factors [78]. Indeed, it has been shown that propionate and butyrate exert an influence on the intracellular potassium level [78] and that butyrate alters the levels of the neurotransmitters GABA, glutamine, and glutamate in the hypothalamus [79]. Thus, several studies have found that the gut microbiome composition and metabolome are altered in many brain disorders, suggesting that SCFAs play an important role in gut–brain axis signaling as any disturbance in this signaling may have direct influence on the CNS and could lead to neurodevelopmental disorders and neurodegenerative diseases [80,81].

On the other hand, the microbiota is important for the electrophysiology of the enteric nervous system (ENS) neurons; for example, germ free mice showed decreased excitability of gut neurons as a result of lower resting membrane potential [82]. Bacteria also affect the function of the vagus nerve; it has been shown that long-term treatment with *Lactobacillus rhamnosus* reduces anxiety and depression related behaviors by inducing region dependent alteration of GABA expression in the brains of mice. Vagotomized mice, however, did not show these neurochemical and behavioral effects, supporting that the vagus nerve is important for communication between the gut microbiota and the brain [83]. Microbiota can communicate with the brain via the vagus nerve and by affecting the circulation of both pro-inflammatory and non-inflammatory cytokines. The systemic circulation of pro-inflammatory cytokines increases BBB permeability resulting in brain inflammatory responses and later cell death [84]. Furthermore, the gut microbiota can mediate postnatal granulocytosis through interleukins, IL17A, and G-CSF dependent regulation [85]. As GSF (glia cell stimulating factor) is important for neurogenesis, microbiota can be used to combat the progression of neurodegenerative diseases [86]. 

As mentioned above, the gut microbiota also indirectly affects the brain through its control over the immune system. This modulation can also occur as a result of gut microbes’ ability to shape T cell responses, affect intestinal antigen presenting cells in the gut associated lymphoid tissues (GALT) like macrophages and dendritic cells, and secrete metabolites that circulate systemically and affect the gene expression of immune regulation as well as autoimmunity [87]. As the gut and GALT immune cells are able to cross the blood brain barrier (BBB), they can affect its neurons and glia [88]. The microbiota metabolites can be neurotoxins, such as clostridial neurotoxins, that prevent neurotransmission through affecting the SNARE complex (Soluble N–ethylmaleimide sensitive factor (NSF) attachment protein receptor); neurotoxins also damage the CNS during infection [89].

The observed effects of probiotics and fecal transplants in animal models or in clinical trials clearly support a role of gut bacteria in modulating cognition and behavior [90]. Bacteria have been shown to produce a large number of metabolites, hormones, and neurotransmitters, including dopamine, norepinephrine, serotonin, or gamma-aminobutyric acid (GABA). While neurotransmitters can unlikely cross the blood brain barrier, which cells do not express necessary transporters, their precursor amino acids and other metabolites like the short chain fatty acids can cross the barrier [91]. In addition to its effect on immunity, several indirect mechanisms have been proposed to explain the effect of gut metabolites, hormones, and neurotransmitters on brain physiology: a) by altering host biosynthesis pathways [91], b) by altering the activity of the stress-associated hypothalamic–pituitary–adrenal (HPA) axis [92], c) by inducing a vagal nerve stimulation [83,93], d) by retrograde transport through the vagus nerve [94]. There is no evidence yet that gut neurotransmitters could cross the blood bran barrier (BBB), however interesting studies showed that gut microbes can directly affect the permeability of the BBB [77]. The blood brain barrier selectively transports some peptides and regulatory proteins such as insulin, leptin, ghrelin, and pancreatic polypeptide all cross the BBB. Insulin is a well-studied example of how a gastrointestinal hormone is able to influence the blood brain barrier. As short-chain fatty acids released in the circulation can regulate the insulin secretion by pancreatic cells, they also indirectly affect its regulation of the blood brain barrier. Indeed, studies showed that insulin can alter the transport rate across the barrier of several substances, including the amino acid tryptophan [95], which is a precursor of serotonin production in the brain and leptin [96], which is involved in feeding behaviors. An important study by Hsiao et al. (2013) [97] reported that intrauterine germ-free mice, displayed an increase in the blood brain barrier permeability and that this increase was associated with a reduced expression of the proteins of the tight junctions (occludin and claudin-5), which are essential for integrity of the barrier. Interestingly, when these germ-free mice were exposed to a normal gut microbiota at the adult age, the tight junction proteins expression were up-regulated, and the permeability of the blood brain barrier decreased. These important results suggest that an early dysbiosis could impair the permeability of the BBB, which can be maintained for long periods of time, but which can also be reversible by the introduction of a normal gut microbiota.

## 3. The Altered Microbiota of People with Autism

Although the main problems associated with autism are cognitive and social skills deficits, it has been shown that gastrointestinal symptoms are widely reported in this cohort [37]. Gut dysbiosis has been reported by numerous studies [23,98,99]. Multiple factors could contribute in dysbiosis in ASD, including the restricted diets that they follow, in response to allergies and gastrointestinal discomfort [39]. As the gut microbiota expands greatly after birth, several investigators explored the possibility of the role of early exposure to antibiotics, either prenatally or at early life, in causing this dysbiosis [100,101]. Indeed, oral antibiotics disrupt the gut microbiota by indirectly promoting the growth of pathogenic microbiota resulting in GIS [100]. A recent study conducted by Strati and colleagues compared the gut microbiota in a group with autism to a neurotypical control group. The study found differences in the Firmicutes/Bacteroidetes ratio explained by the significantly lower levels of Bacteroidetes in the ASD cohort [102]. These significantly low levels of Bacteroidetes are also common to people suffering from inflammatory bowel diseases, pointing to the fact that microbiota dysbiosis is linked to the disruption of homeostasis [103]. This result is also supported by Williams and colleagues in a study that explained the low Bacteroidetes abundance by the expression of the transcription factor CDX2 which, in turn, is associated with the low expression of disaccharides and hexose transporters important for carbohydrate digestion. This study, therefore, suggested a link between genetic expression and microbiota composition in children with autism [104]. However, this is not supported by all studies; a study by Zhang and colleagues found that Chinese children with autism exhibited higher levels of Bacteroidetes. Differences in lifestyle and the absence of a Western diet are cited as contributing factors [105]. Interestingly Kang and colleagues found no significant difference between the Firmicutes/Bacteroidetes ratio in neurotypical and people with autism groups [106]. 

There are some inconsistencies in studies that have investigated the differences in the abundance of certain microbial and fungal strains in individuals with autism and those who are neurotypical (Table S1). As explained by Martínez-González and Andreo-Martínez, this could be due to the different age groups of the participants, their interpersonal differences (like nationality and diet), and different sampling and analytical techniques [107]. 

It has been shown that *Candida Albicans* is found in higher proportions among individuals with autism. Its excessive abundance results in the hampering of carbohydrate and mineral absorption and in higher toxin levels, all of which are thought to contribute to autistic symptoms [108]. An older study by Adams and colleagues (2011) found no significant difference between the yeast content in children with autism and a control group. A high D-arabinitol/L-arabinitol ratio is characteristic of Candida fungal invasion as D-arabinitol is one of its metabolites. Daily administration of *Lactobacillus acidophilus* probiotic has been successful in decreasing this ratio and improving concentration levels and compliance of ASD individuals [109].

Concerning *lactobacilli* species, it has been shown that *L. rhamnosus* and *L. acidophilus* are important for maintaining the intestinal membrane during Shigella infection [110]. Iovene and colleagues found significantly lower amounts of lactobacillus in children with autism using a simple culture-based approach [111]. Building on the results of Moorthy and colleagues, Iovene and colleagues used the results of both studies to explain that lactobacilli are important for maintaining the intestine’s tight junctions, concluding that their presence at low levels resulted in an increase in lactulose absorption in ASD in their own study [111]. However, in a culture independent study by Strati and colleagues (2017) the lactobacilli were higher in ASD subjects [102]. As previously mentioned, this study also found higher levels of *Candida* in ASD subjects, which is inconsistent with the results of Kałużna-Czaplińska and Błaszczyk’s study, which highlighted how lactobacilli can reduce Candida growth [110]. This discrepancy indicates a need for further research in this area.

There are significantly low levels of *Prevotella* in people with autism [112,113]. This can contribute to autistic behavior and/or GI symptoms as it has been shown that *Prevotella* plays important roles by degrading and fermenting polysaccharides, which results in the production of SCFAs [114]. *Prevotella* has also shown to be important in maintaining the community structure in the gut microbiome and in synthesizing vitamin B1 [115,116]. This decrease can be explained by the aforementioned decreases in disaccharide transporters expression. As mono- and disaccharides are not absorbed into the upper GI tract in people with autism, they enter the large intestine and their fermenters outcompete *Prevotella* [106]. A study suggests that this lower level of *Prevotella* can be explained by an altered diet of children with autism [106]. However, their study showed no association between seafood consumption and *Pevotella* levels in people with autism. 

Kang and colleagues (2013) also found that *Veillonellaceae* levels were observed to be lower in subjects with autism [106]. *Veillonellaceae* are known to ferment lactate. Their decrease can partly explain the high levels of lactate observed in people with autism [107]. In another study by Pulikkan and colleagues, however, it was found that when both the control and ASD groups are on an omnivore diet, *Veillonellaceae* levels are abundant in the control and test group but are still significantly higher in people with autism [117]. Again, such conflicting results suggest a need for further investigation. 

Different studies have shown that *Clostridium* is not only significantly more abundant in people with autism but also has an association with constipation and gastrointestinal symptoms. A study conducted by Strati and colleagues shows that *Clostridium* cluster XVIII is more elevated in constipated patients with autism than in non-constipated subjects. Interestingly, no difference in the abundance of *Clostridium* cluster XVIII was found between constipated and non-constipated neurotypical subjects [102]. This is also supported by another study conducted by Paracho and coworkers that shows the fecal content of *Clostridium histolyticum* was higher in children with ASD when compared to unrelated neurotypical controls. The study also shows that raised *Clostridium histolyticum* abundancy is closely associated with the existence of GI problems [118].

Finally, a study by Finegold et. al, 2010 shows high levels of *Desulfovibrio* in participants with autism. This affects the colon’s health since *Desulfovibrio* metabolite hydrogen sulfide contributes to inflammation as it is cytotoxic to epithelial cells [119]. *Streptococcus* and *Coprococcus* have also been shown to have lower levels in people with autism [114,120]. 

## 4. Immunity, Inflammation and ASD

Microbial dysbiosis in autism is an important area of study if a cure for the disorder is to be found. Due to the complex nature of the disorder, existing studies determine associations rather than causal effects. For example, a study by Rose and colleagues compared the microbiota and immune responses of typically developing children with and without GIS, and children with autism with and without GIS [121]. Results showed that, following toll-like receptor stimulation, the autism group with GIS had higher levels of cytokines including IL-5, IL-15, and IL-17 compared to the autism group that were not presenting with GIS. Moreover, it was found that the production of regulatory cytokine TGFβ1 was lower in the autism group with GIS than both groups not presenting with GIS. The same study also found differences in the microbiome composition of children with GIS in the autism and typically developing groups [121]. Similar results were found by Luna and colleagues, who showed that while *Clostridiales* levels were higher in the group with autism and GIS, there were low levels of *Dorea* and *Blautia,* as well as *Sutterella* in this cohort. This dysbiosis correlated with abdominal pain, along with the imbalance of specific cytokines in this group. It was also found that metabolites like tryptophan and cytokine IL6 showed the highest levels in the autism group with abdominal pain [122]. 

Moreover, two studies by Ashwood and colleagues support the existence of mucosal immunopathology in people with ASD where they show that there is a high pro-inflammatory substance circulation and low regulatory circulation. They found that CD3^+^ was more prevalent in the duodenum and colon of children with ASD compared to controls. CD3^+^ TNF α^+^, CD3^+^ IL-2 ^+^, and CD3 ^+^IFNγ^+^, and epithelial CD3 ^+^IL-4 ^+^ were all more common in participants with ASD. On the other hand, CD3 ^+^IL-10 + was less prevalent in participants with ASD when compared with non-disorder control, unlike Crohn and non-Crohn colitis [123,124]. It has also been found that S100A9, a pro-inflammatory advanced glycation end product, level was elevated in people with autism and the expression of its receptor for advanced glycosylation end products (RAGE) was hampered. The advanced glycation end products (AGEs) are known to result in neuroinflammation, oxidative stress and neuronal degeneration and this result shows an affected AGE/RAGE axis in ASD which might result in inflammation [125]. Study results show that treatment with pioglitazone (an anti-inflammatory drug), exerts its effect in glial cells and causes cell death in activated T-lymphocytes. Such treatments also improved autistic signs and symptoms like lethargy, stereotypy, irritability, and hyperactivity without adverse effects. This supports the hypothesis that these symptoms are in part caused by inflammation [126]. Other studies however show no inflammation underlying GIS; for example, a study that looked into fecal calprotectin and rectal nitric oxide, two inflammatory biomarkers, found no indicator of inflammation in participants with ASD; however, this study only had 13 participants so further research is needed [127].

Abnormal immune system function was also reported in people with ASD [128]. As mentioned in a review by Critchfield and colleagues, “Enhanced T cell activation, heightened immunoglobulin and cytokine profiles, as well as histologic changes assessed in intestinal biopsies such as infiltration of lymphocytes, monocytes, natural killer cells and eosinophils have been described in children with autism”. As previously mentioned above, this could be due to dysbiosis. While these results show an association between dysbiosis and impaired immune response, a causal relationship and its direction are yet to be determined. 

## 5. Effect of Probiotics in Restoring the Balance of the GUT Microbiota to Reduce ASD Symptoms

### 5.1. Potential Effect of Probiotics to Treat IBS 

Irritable bowel syndrome (IBS) is a gastrointestinal disorder that is often found in individuals with autism as a comorbidity [129]. Signs and symptoms include diarrhea, constipation, and abdominal discomfort that usually improves after passing stool. Interestingly, anxiety disorders, such as sleep difficulties, anxiety, depression, and headache [129] are also found among people with gastrointestinal diseases, thus illustrating the gut–brain axis relationship. No consensus has yet been reached in finding specific taxonomic prints of these disorders, as taxonomic studies are slowly moving toward metabolomic studies [8,27,130].

Since the role of the gut microbiota in GI diseases is increasingly recognized, interest in interventions that can modulate the microbiota and its interactions with its host have been investigated [131]. When dysbiosis occurs in disorders like IBS and ASD, the combination of diet, prebiotics, and probiotics may represent a low-risk potential therapeutic solution to sustain a healthy microbiome or to restore balance [131,132,133,134]. Although, the exact mechanisms of the effects of probiotics in the human body are not fully understood, it has been shown that probiotic supplements improve IBS and gastrointestinal disorder symptoms through manipulation of the gut microbiota. Many studies supported the hypothesis that probiotics can be used to treat IBS. The outcomes of these studies were sufficient to encourage further future research using the probiotics to treat ASD individuals who also suffer from gastrointestinal disorders [135]. 

There are many studies that tested the benefit of using probiotics to treat irritable bowel disease symptoms. Ford and colleagues conducted a review of 23 randomized controlled trials involving a total of 2575 participants. The aforementioned trials tested the effect of probiotics on IBS against a placebo. An analysis of their results revealed a dichotomous variable; 55.8% of people who received probiotic treatment reported no improvement in their symptoms compared to 73.1% of those who received placebo treatment [136]. In another review by Brenner and colleagues, 16 randomized control trials were considered. Out of these, two that tested the effect of *Bifidobacterium infantis* 35624 probiotic on IBS were the only ones that were both designed properly and yielded favorable results [137]. In the first study of the two, conducted by O’ Mahony and colleagues, 77 patients diagnosed with IBS were given either Lactobacillus salivarius UCC4331, *B. infantis* 35624 1 × 10^10^ CFU/mL formulation, or placebo for 8 weeks. After the 8-week period, assessment showed that the patients who received *B. infantis* 35624 showed significant reduction in cardinal symptoms including abdominal pain or discomfort and distention or bloating. However, bowel movement frequency was not improved [138]. In a larger study conducted by Whorwell and colleagues, 362 IBS female patients were randomized to either a low or high dose of *B. infantis* 35624 or placebo [139]. The results were consistent with O’ Mahony’s study and showed a significant decrease in symptoms like bloating/distention, abdominal pain/discomfort, passing of gas, straining with defecation, sense of incomplete evacuation, and bowel habit satisfaction for the group receiving the 1 × 10^8^ CFU/mL concentration of *B. infantis* 35624 [139]. However, the *B. infantis* 35624 1 × 10^10^ CFU/mL formulation did not confer similar results in the Whorwell study [140]. Brenner and colleagues explained in their review that the reasons why 1 × 10^10^ CFU/mL of *B. infantis* conferred benefits in the O’ Mahony study but not in the Whorwell study may be due to the inclusion of only female participants, stratified randomization, and lack of subjection to type 1 error in the latter study. Furthermore, the 1 × 10^10^ CFU/mL of *B. infantis* formulation was resistant to breakdown in Whorwell’s study [141]. 

Although probiotics have exhibited an effect on IBS symptoms, it has been shown that multi-strain probiotic treatment compared to placebo and mono-strain probiotic treatment has more benefits in alleviating IBS symptoms [132]. However, a major problem with studies reporting the use of probiotics as a safe therapy is their small sample size, which does not enable a conclusion but rather suggests a trend. Finally, studies have shown robust evidence that probiotics are effective for acute infectious diarrhea, antibiotic-associated diarrhea, Clostridium difficile-associated diarrhea, which is one of the problems in ASD, irritable bowel syndrome, and functional gastrointestinal disorders [135,140]. 

### 5.2. The Use of Probiotics in Treating ASD 

Probiotics have proved helpful in readjusting the abundance of bacteria in the gut, thus reducing GIS. Probiotics differ in their efficiency based on how well they survive physio-chemical conditions of the gut, including gastric acid, competition with existing microbiota, and bile secretions [141]. It was found that aerobic probiotics are not necessarily a prominent part of the human gut microbiota and are short-lived in the gut whose microbiota is predominantly anaerobic; thus, the benefits of oral aerobic probiotics are controversial [39]. The mechanisms of successful probiotics include “the excretion of acids (lactate, acetate), competition for nutrients and gut receptor sites, immunomodulation and the formation of specific antimicrobial agents” [141]. 

Because probiotics have been shown to stabilize the intestinal barrier, modulate the immune system, reduce gut inflammation, and ameliorate GI symptoms in IBD models and in children with IBD [74,135,140,142,143,144,145,146,147,148,149,150,151,152], it has been proposed that probiotics may reduce the inflammatory state and modulate the GI and behavioral symptoms in ASD. In fact, probiotics presumably reduce the gut inflammation not only by reducing the gut barrier permeability but also by downregulating the inflammation caused by cytokines as well as other immunomodulatory effects. Various studies have shown that probiotics are able to induce both: 1) the production of pro-inflammatory cytokines in order to alleviate or reduce the gut immune inflammation that is associated with gut dysbiosis, and 2) the production of anti-inflammatory cytokines in order to have a balanced homeostasis by reducing an excessive inflammatory reaction [153,154,155,156,157]. Wang et al. showed that oral probiotic administration during pregnancy prevents autism-related behaviors in offspring by inhibiting the production of proinflammatory interleukin 6 (IL-6) and IL-17a s in both maternal serum and fetal brains. In addition, in vivo administration of the bacterial strain *Lactobacillus rhamnosus* GG has been shown to have anti-inflammatory properties by inducing higher IL-10 serum levels in allergic children [158]. On the other hand, it has been shown that there is bidirectional communication between the gut, the immune system, and the brain. This communication can be manifested for example by 1) the stress that can induce changes on the gut microbiota or by 2) the direct communication between intestinal bacteria with the central nervous system [159]. On the same context, administration of propionic acid in a rat model, a metabolite produced by the intestinal bacteria, has been shown to change both in brain and behavior in a manner comparable with symptoms associated with ASD [160,161]. Moreover, administration of some probiotics can have influences on neuronal function, as shown by different studies. For example, the drink of a probiotic containing *Lactobacillus casei* has revealed positive effects on mood and cognition in volunteers [161]. 

Different studies using animal models with ASD as well as clinical studies in children with ASD examined the use of probiotics as a potential ASD treatment. The probiotic interventions were varied across the pre-clinical and clinical studies and the majority of the supplemented probiotics consisted of *Lactobacillus* species. 

#### 5.2.1. Animal Studies 

There are a number of pre-clinical studies whose results support the positive effect of probiotics on GIS associated with ASD. Table S2 provides a comparison of probiotic supplementation across different pre-clinical studies. For example, research by Liu and colleagues shows evidence that some but not all of the four human-derived probiotic *Lactobacillus reuteri* strains improve lipopolysaccharide (LPS)-induced intestinal inflammation in newborn Sprague Dawley rat pups’ rats. The results also show that these strains had different effects on LPS-induced inflammation by reducing the intestinal level of IL-8 as well as by affecting the intestinal inflammatory cytokine and chemokine production in newborn rats [162]. In fact, it was demonstrated that LPS reduced the intestinal barrier function and the intestinal tight junction permeability [163,164]; thus, it decreases the intestinal histological damage caused by LPS, ameliorates the intestinal permeability, thus by reducing the leaking of endotoxins and inflammatory cytokines through the intestinal barrier. This study demonstrates that human-derived probiotic *Lactobacillus reuteri* strains differentially reduce intestinal inflammation; this suggests that leading to a recommendation that it is appropriate to evaluate different strains of the same probiotic carefully, because they may affect the host differently. In the same context, a study conducted by Wang et al. showed that oral probiotic administration during pregnancy prevents autism-related behaviors in offspring. It has been shown in this study that the administration of 1.5675 × 10^7^ cfu *Bifidobacteria (B. bifidum and B. infantis)* and 5.28 × 10^8^ cfu *Lactobacillus helveticus* every 24 h for 21 days in addition to prebiotics (fructooligosaccharides (FOS) and maltodextrin) decreases the incidence of ASD in offspring and inhibits the production of proinflammatory interleukin 6 (IL-6) and IL-17a, which are key cytokines in the induction of ASD by maternal immune activation [165].

An interesting pre-clinical study into the effect of probiotics on ASD was conducted by Hsiao and colleagues [97]. The study focused on the link between maternal immune activation and autistic behaviors that are reversible with probiotics. It is hypothesized that the outcomes of this study can potentially be generalized to humans. The Hsiao study showed that injecting pregnant mice with an immunostimulant resulted in offspring who showed affected gut barrier integrity evident in the imbalance of metabolites in their serum; the offspring also displayed autistic behavior. The administration of 10^10^ CFU/day of *Bacteroides fragilis* was successful in bringing serum metabolites, gut microbiota composition, and behavioral symptoms back to the normal [97]. These outcomes support a gut microbiome–brain connection in a mouse model with ASD; consequently, the use of Bacteroides strains may be considered as a potential safe therapy for GI and particular behavioral symptoms in ASD. 

A more recent study performed on 50 juvenile hamsters, in which autistic like behaviors were induced by clindamycin and propionic acid (PPA) administration, investigated the effect of the supplementation of a mixture of *bifidobacteria* and *Lactobacilli strains* (ProtexinR). In addition, the administration of these probiotics ameliorated glutamate excitotoxicity through restoring the depleted GABA and Mg^2+^ and decreasing the excitatory neurotransmitter, glutamate [166].

#### 5.2.2. Clinical Studies 

Clinical trials performed mainly on autistic children or children with ASD symptoms to study the effect of probiotics are summarized in Table S3. Among the thirteen clinical studies, two of them were case reports. The first one was related to a 6-year-old boy with ASD and showed that, with the supplementation of probiotics for 8 weeks, there was an improvement in school records and in the attitude against taking a variety of food. In addition, the study showed that the boy’s behavior and situation reversed back when the supplementation with probiotics was stopped [167]. The second case study documents the treatment of a 12-year-old boy with ASD and severe cognitive disability; his diet was supplemented with VSL#3 as a probiotic for 4 weeks followed by 4 months follow up. There was a subsequent reduction in the severity of abdominal symptoms and reduction in neurobehavioral and gastrointestinal symptoms [168]. Although the 2 case studies were temporally separated and used different probiotics, both provide strong evidence that probiotics have a beneficial effect on children with ASD, and are effective in changing their behavior. 

The remaining clinical studies published in the last decade can be divided into two categories based on the criteria of a mono or multi strain approach to probiotic therapy. The first category of clinical study (six studies) provided a single strain probiotic as a supplement [59,109,118,169,170,171]. The second category (five studies), provided multi strain formulations [40,60,172,173]. It was shown that the two, distinct categories of clinical studies presented an important difference in results; this is consistent with results from the meta-analysis by Ford et al. [136]. The studies presented in this review were focused mainly on the investigation of the effect of probiotics on GI symptoms, on behavioral symptoms, and on the gut microbiota. Firstly, it was demonstrated that probiotics interact with the gut microbiota and may downregulate GI inflammation and the intestinal barrier permeability [134]. Secondly, probiotics properties may modulate the inflammatory immune system responses in individuals with ASD, which leads to the improvement of their behavior [174,175]. 

A mono probiotic strain supplementation of *Lactobacillus* was the main common point in all five studies (Table S3). Parracho and colleagues conducted a double-blind placebo crossover trial in the United Kingdom with 22 children with ASD aged between 3–16 years using *Lactobacillus plantarum* WCFS as a probiotic [118]. This study showed no major differences in GI symptoms, but a significant increase in *Lactobacilli/Enterococci* and a decrease in the *Clostridium coccoides* in the stool samples of children with ASD as compared with the placebo group. Moreover, the ASD children showed an improvement in their unsocial behaviors, anxiety, and communication problems [118]. Another study administered *L. acidophilus* as a probiotic supplement to 22 children with ASD aged between 4–10 years with a dose of 5 × 10^9^ CFUs 2 times per day for 8 weeks [109]. The results showed effects comparable to the previous study; this probiotic ameliorated the children’s behavior, improved their ability to concentrate and to follow orders, and showed other changes in ASD symptoms. Kulzna-Czaplinska and Blasczyk did not directly measure the gut microbiota of children with ASD but rather measured the urine D-arabinitol (DA), which is a metabolite biomarker of the Candida species [176]. A decrease was reported in the level of DA and D-/L-arabinotol in the urine of children with autism, leading to a conclusion that the use of *L. acidophilus* as a probiotic changes the microbiota in ASD children by reducing the invasive candidiasis in ASD. A study by Pärtty and colleagues investigated the administration of *L. rhamnosus* GC (ATCC 53103), another lactobacillus species, to 75 infants for the first 6 months of life. The cohort was followed for 13 years and exhibited a reduced risk of neuropsychiatric disorder development later in childhood. One mechanism cited was a change in gut microbiota composition [169]. Furthermore, this study showed that at the age of 13 years, 6 out of 35 children who took the placebo were diagnosed with ASD or attention-deficit hyperactivity disorder (ADHD), while none in the probiotic group developed these conditions. Again, this provides compelling evidence for the efficacity of lactobacillus as probiotic to modulate some symptoms in ASD. Slykerman and colleagues administered a probiotic strain; *Lactobacillus rhamnosus* HN001 or *Bifidobacteria animalis* subsp. lactis HN019, to a group of 342 children who were subsequently followed from birth to 11 years in New Zealand. The cohort demonstrated no associated neurocognitive outcomes at 11 years of age [59]. Although this study did not report any beneficial effects, Slykerman et al. did not exclude the fact that other probiotics may have beneficial effects on ASD. Finally, a pilot study combining both probiotic and prebiotic as supplementation showed a reduction in the frequency of certain GI symptoms, as well as reduced occurrence of particular aberrant behaviors in children aged 2–11 with ASD and GI co-morbidities [171]. Megan et al. showed that the administration of *Bifidobacterium infantis* in combination with a bovine colostrum product (BCP) reduced IL-13 and TNF-α production in some participants [171]. 

The multi-strain probiotics supplemented in the five studies were all different from each other, however combinations included *Lactobacillus* and *Bifidobacterium* species (Table S3). Moreover, the types of probiotic supplements administered in the trials were different in terms of form, dose, bacterial strains, and combinations of bacterial strains. All these studies showed beneficial effects, including significant improvements in GI symptoms. West and colleagues [173] administered a cocktail of *Lactobacillus delbrueckii*, *L. acidophilus*, *Lactobacillus casei*, *B.longum*, and *Bifidobacteria bifidum* to 33 children with ASD aged between 3–16 years for 21 days. They observed a subsequent reduction in severe constipation from 52% to 20% and in diarrhea from 20% to none. Tomova and colleagues [40] found that 90% of the 10 children with ASD had some form of GI symptoms. The severity of the GI symptoms was directly proportional to the severity of the ASD. In addition, higher levels of fecal TNF-α were observed in children with ASD and their siblings compared with children without ASD. In fact, the correlation between TNF-α concentrations and GI symptoms was used as a marker for intestinal inflammation [40]. Similarly, the use of a multi-strain cocktail composed of *Lactobacillus acidophilus*, *Lactobacillus rhamnosus*, and *Bifidobacteria longum* 1 time/day for 3 months was investigated by Shaaban and colleagues [172]. This study discovered a significant improvement in GI signs and symptoms including constipation, stool consistency, flatulence, and abdominal pain after three months of probiotic intake. 

Two of the five studies evaluating the effect of a supplement with multi strain probiotics found significant modulation of the gut microbiota [40,172]. The Tomova study used real time PCR to analyze fecal microbiota, and found a decrease in the level of *Bacteroidetes/Firmicutes* ratio and an increase in the level of Lactobacillus in children with ASD as compared with controls [40]. Similarly, a decrease in *Desulfovibrio spp*, a biomarker for GI disturbance of autistic patients, in the stool was observed [31]. Likewise, Shabaan and colleagues found an increase in Bifidobacterial and Lactobacilli levels after 3 months supplementation with the probiotic [172]. 

As described previously, ASD individuals may show some maladaptive behaviors linked to the GI dysfunction. As such, two of the five clinical studies summarized in Table S3 examined change in behavior after probiotic supplementation. West and colleagues [173] and Shabaan and colleagues [171] found an improvement in the Autism Treatment Evaluation Checklist score (ATEC) for their participants. In fact, the ATEC is used as a test to evaluate autism severity across 4 domains: language/speech/communication, sociability, sensory/cognitive awareness, and health and physical behavior [177]. Lower ATEC scores indicate less severe ASD symptoms. In the West study [173], the ATEC score decreased from 72.8% to 58.3% after treatment with probiotics. Similarly, the Shabaan study [172] also found a decrease in ATEC scores, the biggest improvement being in the health and physical behavior category which decreased from 36.83% to 27.1%. Finally, a study done on thirteen children aged from 3 to 12 years old and who were suffering from ASD, anxiety, or GIS showed that that the administration of the Visbiome formulation, containing 8 different strains mainly lactobacillus, was safe and provided health improvements [178]. Altogether, the evidence presented here supports the continued investigation of different probiotics in preventing and managing ASD; this may explain the multiple new, ongoing, clinical human trials using probiotics to prevent/treat ASD, which are summarized in Table S4.

Although the types of probiotic supplements administered in the trials were different in terms of form, dose, bacterial strains, and combinations of bacterial strains, all these studies showed beneficial effects, including significant improvements in GI symptoms. Moreover, comparing studies using mono strain probiotic supplements and studies using multi-strain probiotic supplements, shows a better effect in alleviating ASD symptoms when using multi strain probiotics. Of note, the range of methodologies used in the different clinical trials makes comparisons difficult; this hinders a clear conclusion but does, however, reveal a clear trend. For example, the trials differ greatly in sample size; furthermore, homogeneity of the patients in terms of demographics, symptoms, and dietary habits is lacking. There are also inconsistencies in strains, dose, and duration of probiotic intervention that limit the possibility of identifying which probiotic/species/ strain or therapy has contributed the most to improvements in ASD symptoms. Despite the above investigative problems, the use of probiotics in clinical studies to modulate GI dysfunction and ASD, yields promising results. More randomized, controlled studies with a larger population size and homogenized methods of DNA extraction may lead to more robust studies and results. 

## 6. Conclusions

A dramatic increase of the prevalence of ASD worldwide was noticed during the last decade. Several approaches are being studied to treat various symptoms of ASD, which include both pharmacological and non-pharmacological treatments [179]. Indeed, stem cell transplantation shows promising results on inflammation and improvements for some symptoms; cognitive behavioral therapy is used for repetitive behaviors; music therapy is used for cognitive, speech, and motor training [180,181]. 

Research has determined that a large percentage of individuals with ASD also suffer from GIS, and a growing number of studies point at a correlation between the two [179]. An altered gut microbiota was found in individuals with ASD [8,27,179]. 

The modulation of the gut microbiome can be done either by using fecal transplants [182] (or the transplant of fecal samples from a healthy individual to a non-healthy one) or by the means of lifestyle interventions [183]. While fecal transplants have shown promising results on ASD, they only aim at *treating* dysbiosis and symptoms, while lifestyle changes such as dietary intervention address the *causes* of dysbiosis, thereby allowing for both *prevention* and *treatment*.

In this review, we attempted to describe the benefits of targeting the gut microbiome using probiotics as a novel approach to tackle both the comorbidities linked to ASD, such as gastrointestinal disorders and inflammation, and the cognitive issues via the interactions of the Gut-Brain axis. We described the important potential benefits of using probiotics as a way to upgrade the microbiome, in order to provide with necessary metabolites that were shown to be beneficial for the gut permeability, immunity, and brain function. It was recently shown by El Tokhi et al. (2020) [9] that the synaptic pruning in patients with neuropsychiatric disorders was found to be dysregulated and this was associated with a dysbiosis. Dietary changes such as diet and probiotics were proposed to improve brain function via a mechanism of microglial-induced synaptic pruning and the formation of new synapses, thereby improving neural function [9]. 

**Figure 2:**
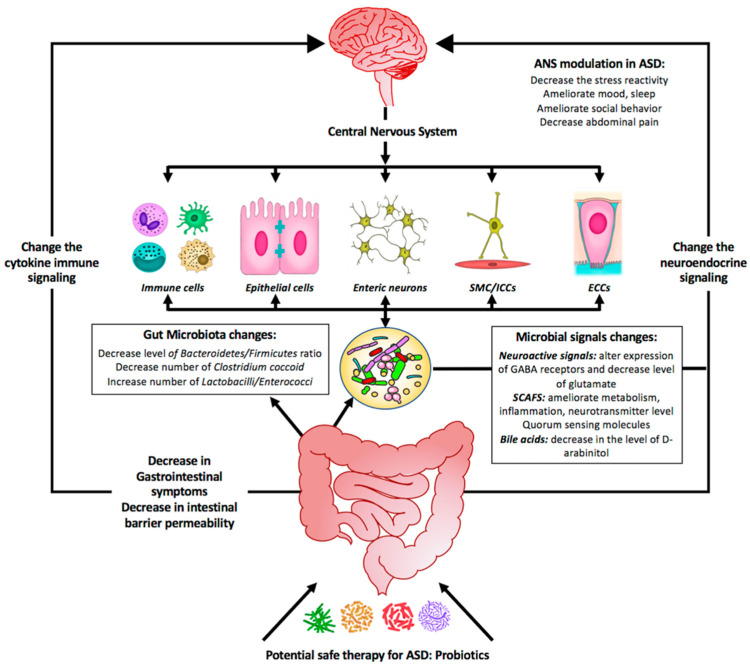
Summary diagram illustrating the effect of probiotics as a potential safe therapy in treating ASD symptoms. Probiotics may colonize the gut and shift the bacterial populations within this system to the so-called good bacteria. Probiotics include supplementation of bacterial-produced amino acids SCFAs (short-chain fatty acids) and other metabolites that might be beneficial by (1) affecting the GI symptoms, (2) affecting the gut microbiota population, and (3) changing the microbial signals. All these will change the neuroendocrine signaling as well as the cytokine immune signaling. As a consequence, the central nervous system will be affected and most of the neurological ASD symptoms will be alleviated.

Numerous studies support the role for probiotics in treating ASD as summarized by Figure 2. Although probiotics show promise in correcting gut dysbiosis and are also yielding favorable results in treating autistic behavior-related symptoms, standardized clinical studies may lead to more robust results and outcomes. By re-establishing a balanced microbial composition with subsequent balanced metabolites secretion, probiotics appear to be an important dietary component and a potentially side effect free therapy that can be proposed to treat GIS and ASD symptoms, by correcting the dysbiosis, reducing inflammation, and reinforcing depleted immunity. 

Nevertheless, it is important to highlight that other dietary components are important for the gut microbiota homeostasis, such as vitamins, short chain fatty acids, polyphenols, and a growing number of studies are currently trying dietary interventions for ASD [179,184]. Studies described nutrition as having a significant impact on the gut microbiome: chemicals, pesticides on food, artificial sweeteners or preservatives have been shown to disrupt the biodiversity and function of the gut [185,186]. The gluten free/casein free diet, the Atkins diet, the DASH diet, the ketogenic diet, specific carbohydrate diet, the Paleo diet are the main diets that have been studied so far for ASD and showed various levels of efficacy [186,187]. All these diets have a common point of excluding processed food and including more dietary fibers that will act as prebiotics and fermented food to promote the shift of the dysbiosis toward eubiosis. 

## Supplementary Materials

Supplementary materials can be found at https://www.mdpi.com/1422-0067/21/11/4159/s1. Table S1: A summary of the altered gut flora in individuals with autism spectrum disorder (ASD); Table S2: Summary of major animal studies of probiotic interventions, their mechanism of action and their outcomes related to ASD; Table S3: Summary of major clinical studies of probiotic interventions, their mechanism of action and their outcomes related to ASD; Table S4: Summary of ongoing human studies of probiotic interventions to ASD.
