# Supplementary material for: The Promising Role of Probiotics in Managing the Altered Gut in Autism Spectrum Disorders
Source: Int J Mol Sci. 2020 Jun 10;21(11):4159. doi: 10.3390/ijms21114159 (PMC7312735; doi:10.3390/ijms21114159)
Supplement: Supplementary file 1 [file ijms-21-04159-s001.pdf]

## Supplementary Materials for:

# The Promising Role of Probiotics in Managing the Altered Gut in Autism Spectrum Disorders

**Basma Abdellatif[1], Clare McVeigh[2], Ghizlane Bendriss[3] and Ali Chaari*[4]**

[1] Premedical department, Weill Cornell Medicine, Qatar Foundation, Education City, P.O. Box 24144, Doha, Qatar.; bwa4001@qatar-med.cornell.edu
[2] Premedical department, Weill Cornell Medicine, Qatar Foundation, Education City, P.O. Box 24144, Doha, Qatar.; clm2021@qatar-med.cornell.edu
[3] Premedical department, Weill Cornell Medicine, Qatar Foundation, Education City, P.O. Box 24144, Doha, Qatar.; ghb2002@qatar-med.cornell.edu
[4] Premedical department, Weill Cornell Medicine, Qatar Foundation, Education City, P.O. Box 24144, Doha, Qatar.; alc2033@qatar-med.cornell.edu

* Correspondence: alc2033@qatar-med.cornell.edu; Tel.: +974 33593586

**Table S1.** A summary of the altered gut flora in individuals with autism spectrum disorder (ASD).

| ***Bacteria/Fungi*** | **Status in ASD cohorts (compared to neurotypical cohorts)** | **Significance** | **Reference** |
|---|---|---|---|
| ***Candida*** | Higher | Secrete D- arabinitol<br>Affects carbohydrate, and mineral absorption and Secretes toxin | [109, 110] |
| | Same | | [101] |
| ***Lactobacillus*** | Lower | Normally maintain intestines' tight junctions. | [112] |
| | Higher | | [103] |
| ***Prevotella*** | Lower | Normally<br>degrades polysaccharides, important for community structure and vitamin B1 synthesis | [113] |
| ***Veillonellaceae*** | Lower | It ferments lactate | [108] |
| | Higher | The study used an omnivores diet | [118] |
| ***Clostridium*** | Higher | Directly correlated to GI problems | [103] |
| ***Desulfovibrio*** | Higher | Secrete hydrogen sulfide | [120] |
| ***Streptococcus and Coprococcus*** | decreased | | [106] |

**Table S2.** Summary of major animal studies of probiotic interventions, their mechanism of action and their outcomes related to ASD.

| Name of Probiotic strains | Study type | Probiotic type and dose | Duration of intervention | Mechanism of action | Outcomes | Reference | year |
|---|---|---|---|---|---|---|---|
| Human-derived probiotic *Lactobacillus reuteri* strains (*L. reuteri* strains DSM17938, ATCC PTA4659, ATCC PTA 5289, and ATCC PTA 6475) | Newborn Sprague Dawley rat pups | $10^6$ CFU.g body wt$^{-1}$.day$^{-1}$. | 3 days | - Decreased level of IL13 and IFN- level in intestine by *L.reuteri* strain DSM 17938<br>- Decreased level in Lipopolysaccharides (LPS) induced TNF- and IL-1<br>- Decreased of intestinal histological damage produced by LPS<br>- Affect Th1 type and Th2 type cytokines in rat intestine | - Human-derived probiotic *Lactobacillus reuteri* strains differentially reduce intestinal inflammation<br>- **Recommendations:** It is appropriate to evaluate different strains of the same probiotic carefully, because they may affect the host differently | (165) | 2010 |
| *Bacteroides fragilis* NCTC 9343 | Pregnant C57BL/6N mice | $10^{10}$ CFU/ day | 6 days | - Increased in expression of CLDNs 8 and 15 and restoration of colonic IL-6 levels linked to the correction of the gut barrier integrity and permeability in MIA offspring but also correction in tight junction and cytokine expression<br>- Specific commensal intestinal microbiota restored in MIA offspring | - It restored affected gut barrier evident by bringing serum metabolites, gut microbiota composition and behavioral symptoms back to normal in rat pups<br>- Support a gut microbiome-brain connection in mouse model with ASD and the used probiotic may be considered as potential safe therapy for GI and particular behavior symptoms in ASD | [98] | 2013 |

| | | | | | | | |
|---|---|---|---|---|---|---|---|
| | | | | - Corrects ASD related behavioral and neurological abnormalities<br>- Corrects level of production of serum metabolites involved in key metabolism pathways<br>- | | | |
| Mixture of *bifidobacteria* and *Lactobacilli* strains (ProtexinR) | 50 young male golden Syrian hamsters weighing between 60 and 70 g (7 weeks of age) in which autistic like behaviors were induced by clindamycin and propionic acid (PPA) administrati on | 2 × $10^8$ CFU/day | 27 days | - Changes in the gut microbiota<br>- ameliorates glutamate excitotoxicity through increasing depleted GABA and $Mg^{2+}$ and decreasing the excitatory neurotransmitter, glutamate. | - *Bifidobacteria* and *Lactobacilli* supplementation can be suggested as a strategy to ameliorate the glutamate excitotoxic effects of PPA and clindamycin.<br>→ Probiotic treatment reduces the autistic-like excitation/inhibition imbalance in juvenile hamsters | [167] | 2018 |
| Probiotics Sachet Children’s Formula (Biostime, Guangzhou, China): 1.9 × $10^8$ cfu/g | C57BL/6J female mice | 1.5675 × $10^7$ cfu Bifidobacteria (B. bifidum | 21 days | - Increases in IL-6 and IL-17 levels in both maternal serum and fetal brain<br>- Decreases in γ-aminobutyric acid levels in | *Oral probiotics during pregnancy may be an effective means for decreasing the incidence of ASD in offspring* | [166] | 2019 |

| | | |
|---|---|---|
| *Bifidobacteria (B. bifidum and B. infantis*) and 6.4 × $10^9$ cfu/g *Lactobacillus helveticus*, in addition to fructooligosaccharides (FOS) and maltodextrin | and B. infantis) and 5.28 × $10^8$ cfu Lactobacillus helveticus/24h | the prefrontal cortex of adult offspring<br>- Prevents maternal immune activation (MIA)-induced repetitive behaviors in adult offspring<br>- Prevents MIA-induced anxiety-like behavior in adult offspring<br>- Prevents MIA-induced social deficits in adult offspring<br>- Prevents MIA-induced decreases in GABA levels in the PFC of adult offspring |

**Table S3.** Summary of major clinical studies of probiotic interventions, their mechanism of action and their outcomes related to ASD.

| Name of Probiotic strains | Study type | Probiotic type and dose | Duration of interventio n | Outcomes | Reference | year |
|---|---|---|---|---|---|---|
| General probiotics | Case report of 6-years-old boy with ASD | Not mentioned | 8 weeks | - Improvement in school records and attitude against taking a variety of food<br>- His behavior and situation reversed back when the supplementation with probiotics was stopped | [168] | 2000 |
| *Lactobacillus plantarum WCFS1* | Double blind placebo crossover trial in United Kingdom with 22 children with ASD aged between 3 and 16 years old (91 % males) | $4.5 \times 10^{10}$ CFUs daily | 3 weeks | - Increased the number of *Lactobacilli/Enterococci* and decreases the *Clostridium coccoides* found in the stool samples of children with ASD as compared with placebo<br>- Improvement of the anti-social behaviors, anxiety and communication problems<br>- No major differences In GI symptoms | [119] | 2010 |
| *L. acidophilus* | Noncontrolled trial in Poland of 22 children with ASD aged between 4-10 years (90% males) | $5 \times 10^9$ CFUs 2 times per day | 8 weeks | - Decrease in the level of D-arabiinitol and D-/L-arabinotol in the urine of children with autism<br>- Changes in ASD symptoms<br>- Improvement in the ability to concentrate and to carry orders | [110] | 2012 |
| Delpro® (*Lactocillus acidophilus, Lactobacillus casei, Lactobacillus delbruecki, Bifdobacteria longum, Bifidobacteria bifdum*) and Del Immune V® (*Lactobacillus rhamnosus* V lysate) | Noncontrolled trial in USA of 33 children with ASD aged between 3-16 years | 10 billion CFUs total. 1 capsule, 3 times/d for 21 days | 21 days | - Improvement in Autism Treatment Evaluation Checklist score (ATEC) for 88% of children<br>- Improvement in GI symptoms, mainly reported a decrease in constipation and diarrhea, | [174] | 2013 |
| "Children Dophilus": Blend of:<br>-3 strains of *Lactobacillus* (60%), | Noncontrolled trial in Slovakia of 29 Children: 10 ASD, 9 non-ASD siblings, | Not provided | 3 times/day for 4 months | - Decreased levels of *Bacteroidetes/Firmicutes* ratio | [39] | 2015 |

| | | | | | | |
|---|---|---|---|---|---|---|
| -2 strains of *Bifidumbacteria* (25%),<br>-1 strain of *Streptococcus* (15%) (exact strain information not provided) | 10 non-ASD controls aged between 2–9 y: non-ASD siblings = 5–17 y; non-ASD controls = 2–11 y | | | - Increase in the level of *Lactobacillus* in children with ASD as compared with controls<br>- Increased level of<br>- Increased in level of fecal TNF-$\alpha$ in children with ASD and their siblings compared with children without ASD, and there was a strong correlation between TNF-$\alpha$ concentrations and GI symptom | | |
| *L. rhamnosus* GC (ATCC 53103) | Randomized trial, placebo controls in Finland of 75 infants for the 6 months of life and followed for 13 years starting at the age of 6 months | Not provided | The 6 first months of life | - At the age of 13 years, 6 out of 35 children who took placebo were diagnosed with ASD or attention-deficit hyperactivity disorder (ADHD), but none in probiotic group.<br>- Probiotic supplementation early in life may reduce the risk of neuropsychiatric disorder development later in childhood by many mechanisms including the change in gut microbiota composition. | [170] | 1015 |
| VSL#3 (VSL Pharmaceuticals, Ft Lauderdale, FL, USA) : $3\times10^{11}$ CFU/g of bifidobacteria (*B. longum*, *B. infantis* and *B. breve*), $8\times10^{10}$ CFU /g of lactobacilli (*L. acidophilus*, *L. casei*, *L. delbrueckii subsp. L. bulgaricus* and *L. plantarum*) and $20\times10^{10}$ CFU /g of *Streptococcus salivarius subsp. thermophilus*. | Case report of 12-years old boy with ASD and severe cognitive disability | Not provided | 4 weeks followed by 4 months follow up | - Reduction in the severity of abdominal symptoms<br>- Reduction in neurobehavioral and gastrointestinal symptoms | [169] | 2016 |
| Vivomixx probiotic: *Streptococcus thermophilus*, *Bifidobacterium breve*, *Bifidobacterium longum*, | Randomized, placebo-controlled trial of a group of 100 preschoolers with | Dietary Supplement: Vivomixx® | 5 months | - Changes in severity level of ASD and GI symptomatology<br>- Changes in Behavioral Profiles<br>- Changes in Parental Stress | [59] | 2016 |

| | | | | | | |
|---|---|---|---|---|---|---|
| *Bifidobacterium infantis, Lactobacillus acidophilus, Lactobacillus plantarum, Lactobacillus paracasei, Lactobacillus delbrueckii subsp. bulgaricus* | ASD aged between 18-72 months in Italy. Blind randomized 1:1 to regular diet with probiotic or with placebo for 5 months | Two packets (900 billions of bacteria) per os (P.O.) daily x 1 month and one packet (450 billions of bacteria) P.O. daily x 5 months Dietary Supplement: PlaceboTwo packets ( 4,4 grams of maltose and silicon dioxide x 2) P.O. daily x 1 month and one packet (4,4 grams of maltose and silicon dioxide) P.O. daily x 5 months | | - Changes in Electroencephalogram obtained parameters<br>- Changes in levels of serum Lipopolysaccharide, leptin, serum resistin, TNF , IL-6, PAI-1, fecal calprotectin | | |
| 3 strains: *Lactobacillus acidophilus, Lactobacillus rhamnosus, Bifidobacteria longum* | Prospective, open label noncontrolled trial in Egypt of 60 Children: 30 ASD and 30 age-/sex-matched controls (relatives) aged between 5-9 years old | 5 g of powder/day (each gram contained 100x $10^6$ CFUs of each strain) | 1 time/day for 3 months | - Increase in *Bifidobacterial* and *Lactobacilli* levels<br>- Improvement in the severity of autism assets by the decrease in ATEC score<br>- Improvements in GI problems the probiotic has beneficial effect on both behavioral and GI manifestations of ASD | [173] | 2018 |

| | | | | | | |
|---|---|---|---|---|---|---|
| | | | | Could be recommended for children with ASD as an adjuvant therapy | | |
| *Lactobacillus rhamnosus* HN001 *Or* *Bifidobacteria animalis subsp. lactis* HN019 | Two centers, randomized, double blind, placebo-controlled study of 342 children followed from birth to 11 years in New Zealand | Not provided | Mothers given probiotics from 35 weeks pregnant until 6 months. Children receive the treatment from birth to 2 years | - HN001 and HN019 given in early life were not associated with neurocognitive outcomes at 11 years of age.<br>- Recommendations: the study cannot exclude that other probiotics may have beneficial effect. | [59] | 2018 |
| *Bifidobacterium infantis in combination with a prebiotic bovine colostrum product (BCP)* | A double-blind, crossover, randomized clinical trial with children ages 2–11 with ASD and GI co-morbidities | A dose of 4 x $10^9$ CFU twice daily of probiotics and 0.15 g/lb body weight per day for the prebiotics | 12-week study included 5 weeks of probiotic-prebiotic supplementation, followed by a two-week washout period, and 5 weeks of prebiotic only supplementation | - Reduction in the frequency of certain GI symptoms<br>_ Reduced occurrence of particular aberrant behaviors<br>- Reduction in IL-13 and TNF-α production in some participants<br>Although there is some improvements in ASD children's, further research into the efficacy of these treatments should be done.<br>, | [172] | 2019 |

| | | | | | | |
|---|---|---|---|---|---|---|
| VISBIOME probiotic: *Streptococcus thermophilus, Bifidobacterium breve, Bifidobacterium longum, Bifidobacterium infantis, Lactobacillus acidophilus, Lactobacillus plantarum, Lactobacillus paracasei, Lactobacillus delbrueckii subsp. bulgaricus* | Randomized crossover trial with 13 children | Not mentioned | 19 weeks | - The VISBIOME formulation was safe and suggested a health benefit in children with ASD and GI symptoms who retained *Lactobacillus* | [174] | 2020 |
| *L. reuteri* (BioGaia) | Two staged, randomized, double blind placebo controlled, parallel group study of 60 patients with ASD aged between 3-25 years old in USA. | Dietary Supplement: oral probiotics 200 million cfu per day for 24 weeks<br>Dietary Supplement: oral placebo 2 pills per day for 24 weeks | 24 weeks | - Social communication and behavior test<br>- Neuroinflammation and Oxytocin levels<br>- structural and functional MRI<br>- 16s metagenomic sequencing of the microbiome<br>- Vital indices measurements<br>- Assessment of GI function and gut microbiome/SCAFs | [171] | 2020 |

**Table S4.** Summary of ongoing human studies of probiotic interventions to ASD.

| NCT No. | Trial name | Interventions | Probiotic type. dose and | Intervention Model | Outcomes measures | Estimated enrollment | Sponsors | Status |
|---|---|---|---|---|---|---|---|---|

| | | | **duration of intervention** | | | | | |
|---|---|---|---|---|---|---|---|---|
| NCT03514784 | Combination Probiotic:<br>BB-12 With LGG (Different Doses) in Treating Children With Autism Spectrum Disorder | Drug: BB-12 with LGG (Higher Dose)<br>Drug: Placebo<br>Drug: BB-12 with LGG (Lower Dose) | Drug: BB-12 with LGG (Higher Dose: 1o billion CFUs)<br>Drug: Placebo Maltodextrin<br>Drug: BB-12 with LGG (Lower Dose; 1 billion CFUs)<br>Over 56-day period and a 28-day observation period | Blinded randomized controlled study | - Effects of BB-12+LGG at different doses on GI symptoms<br>- Effects of BB-12+LGG at different doses on gut inflammation (S1009A)<br>- Effects of BB-12+LGG at different doses on gut inflammation (Fecal Calprotectin, plasma Zonulin and IL-8)<br>- Using 16S rDNA analysis, this will determine microbial alpha and beta diversity and distribution of the major taxa before and after probiotic treatment<br>- Effects of BB-12+LGG at different doses on metabolites to determine levels of metabolic markers which are reported to be abnormal in autism: fecal amino acids, ammonia, short chain fatty acids (SCFA), and phenols. | 70 healthy children with confirmed ASD status | UTHealth Houston, Texas, United States | Recruiting |

| | | | | | | | | |
|---|---|---|---|---|---|---|---|---|
| NCT02903030 | Efficacy of Vivomixx on Behavior and Gut Function in Autism Spectrum Disorder | Dietary Supplement: Vivomixx Dietary Supplement: Placebo | Probiotic: multi strain probiotic Vivomixx containing 450 billion lyophilized bacterial cells per sachet belonging to 8 probiotic strains. Placebo: 4.4 grams of maltose and silicon dioxide per sachet Duration: 3 months supplementation | Randomized controlled cross-over design with 4-week washout | - A change in the Autism Treatment Evaluation Checklist (ATEC) total score compared to baseline.<br>- A change in the frequency of gastrointestinal symptoms compared to baseline, as measured by the Gastrointestinal History (GIH) questionnaire.<br>- A change in the Aberrant Behavior Checklist (ABC) total score compared to baseline.<br>- A change in the Autism Parenting Stress Index (APSI) score compared to baseline. | 82 children with Autism Spectrum Disorders and co-morbid gastrointestinal symptoms | Ohio State University Wexner Medical Center Columbus, Ohio, United States | Recruiting |
| NCT02903030 | Probiotics for Quality of Life in Autism Spectrum Disorders | Drug: BB-12 with LGG Drug: Placebo | Drug: combination probioticBB-12 with LGG orally (LGG dose: $10^9$ c.f.u.'s; BB-12: $10^9$) for a total of 56 doses Pplacebo (maltodextrin) at 2:1 ratio. Duration: 84 days | Randomized | - Children with ASD & GI symptoms differ in microbiome composition and function from neurotypical children with GI symptoms<br>- Visbiome Extra Strength, improves GI and pain symptoms, correlating with altered gut microbiome | 13 | Ohio State University Wexner Medical Center Columbus, Ohio, United States | Completed Has Results |

| | | | | | | | | |
|---|---|---|---|---|---|---|---|---|
| | | | | | composition and related metabolites<br>- Change in The Aberrant Behavior<br>- Change in Social Responsiveness Scale | | | |
| NCT02674984 | Road to Discovery for Combination Probiotic BB-12 With LGG in Treating Autism Spectrum Disorder | Drug: BB-12 with LGG<br>Drug: Placebo | Drug: Visbiome Extra Strength<br>It is a mix of 8 strains of beneficial bacteria that should improve gut flora, improving GI function and hopefully anxiety.<br>Drug: Maltose (placebo)<br>Maltose with a trace amount of silicon dioxide<br>Other Name: Maltose with silicon dioxide<br>Duration: 8 weeks per condition with 3 weeks washout between | Randomized | - Effect of combination probiotic BB-12+LGG on adverse events assessed by case report form (survey) that are related to BB-12 with LGG (health-promoting bacteria) or placebo treatment.<br>- Effect of combination probiotic BB-12+LGG on irritability and maladaptive behaviors with Aberrant Behavior Checklist (ABC)<br>- Effect of combination probiotic BB-12+LGG on irritability and maladaptive behaviors measured with the Social Responsiveness Scale-2 | 29 healthy children with autism spectrum disorder aged 4-15 years | University of Texas Health Science Center at Houston Houston, Texas, United States | Completed |

| | | | | | | | | |
|---|---|---|---|---|---|---|---|---|
| NCT04293783 | Randomized Double-blind Clinical Trial With *L.Reuteri* Supplementation in Children With Autism Spectrum Disorder | Dietary Supplement: *L.Reuteri* Dietary Supplement: Placebo | Dietary Supplement: *L.Reuteri* daily supplementation with two tablets for six months Other Name: Gastrus Dietary Supplement: *L.Reuteri* daily supplementation with two tablets for six months Other Name: Gastrus | Double-blind randomized, parallel-group, placebo-controlled study | - Changes in GI and ASD symptoms<br>- Changes in Adaptive Functioning<br>- Changes in Behavioral Profiles and parental stress Changes in Metabolomic Profile such as neurotransmitter metabolism, gastrointestinal alterations or dysbiosis, and mitochondrial dysfunction<br>- The effect of probiotic administration on the metabolome will be investigated.<br>- Changes in inflammatory profile (zonulin, IL-17 profile) | 80 children With Autism Spectrum Disorder | University of Bari Bari, BA, Italy University of Rome Tor Vergata Rome, RM, Italy | Recruiting |
| NCT03982290 | Psychophysiological Effects of *Lactobacillus Plantarum* PS128 in Preschool Children With Autism Spectrum Disorder | Probiotic, Lactobacillus plantarum PS128 Placebo: microcrystalline cellulose | Probiotic, *Lactobacillus plantarum* PS128 PS128 capsules, 2 capsules per day Other Name: PS128 Placebo, microcrystalline cellulose | Double-blinded, randomized, placebo-controlled | - Changes from baseline of anxiety subscale in Children's Behavior Checklist (CBCL)/Achenbach System of Empirically Based Assessment (ASEBA) at week 8 and week 16<br>- Changes from hyperactivity subscale of | 250 Preschool Children with Autism Spectrum Disorder | Department of Pediatric Neurology, MacKay Children's Hospital Taipei, Taiwan | Recruiting |

| | | | | | | | | |
|---|---|---|---|---|---|---|---|---|
| | | | Placebo capsules, 2 capsules per day Duration: 16 weeks | | Attention-Deficit/Hyperactivity Disorder Test (ADHDT) at week 8 and week 16<br>- Gut and oral microbiota analysis<br>- Changes in constipation, diarrhea, stool consistency, stool smell, flatulence, and abdominal pain<br>- Changes in Communicative and interactive assessment | | | |
| NCT03858816 | Efficacy of Multiple Strain Probiotics Reduces the Neurobehavioral Disorder in Premature Very Low Birth Weight Infants | Mixture probiotics Other: Placebo | The mixture probiotics capsule: contain 1 ×$10^9$ CFU/1 capsule of mixture probiotics, taking 1 probiotic capsule for up to 4 months after birth.<br>The placebo contains the same excipient ingredients but without the live bacteria: 1 placebo capsule | Double blind, randomized, controlled trial | - Incidence rate of death or ADHD and ASD<br>- Incidence rate of NEC ≧ stage 2, sepsis, severe (grade 3-4) IVH, BPD, liver function and adverse effects or intolerance and neurodevelopment impairment. | 320 Preterm infants ≧ 23 weeks and ≦ 32 weeks gestational age and birth weight below 1500 gm and who survive to NICU | Department of Pediatrics, Children Hospital, China Medical University Taichung, Taiwan | Recruiting |

| | | | | | | | | |
|---|---|---|---|---|---|---|---|---|
| | | | for up to 4 months after birth | | | | | |
| NCT02086110 | Effect of Milk Oligosaccharides and Bifidobacteria on the Intestinal Microbiota of Children with Autism | Milk Oligosaccharides (prebiotic) and Bifidobacteria (probiotic) | oligosaccharides in two divided doses per day orally<br>Prebiotic: bovine colostrum, bovine oligosaccharides administered orally twice per day for a daily total of 0.3 g per pound of body weight for the first five weeks, followed by a two-week break with no treatment, and then will receive the synbiotic<br>Probiotic: *Bifidobacterium infantis* SC268 (10 billion CFU) twice a day plus 0.3 g per pound body weight of bovine milk for | Randomized | - Stool Microbiota Composition Change During Prebiotic Only Treatment<br>- Stool Microbiota Composition Change During Synbiotic Treatment<br>- Serum Immune Profile Change During Prebiotic Only Treatment | 11 children with Autism | UC Davis MIND Institute Sacramento, California, United States | Completed Has Results |

| | | |
|---|---|---|
| | the next five weeks<br>Duration: 8 weeks | |
